# Ultrasensitive Flexible Thermal Sensor Arrays based on High‐Thermopower Ionic Thermoelectric Hydrogel

**DOI:** 10.1002/advs.202302685

**Published:** 2023-07-03

**Authors:** Yang Han, Haoxiang Wei, Yanjun Du, Zhigang Li, Shien‐Ping Feng, Baoling Huang, Dongyan Xu

**Affiliations:** ^1^ Department of Mechanical and Automation Engineering The Chinese University of Hong Kong Shatin, New Territories Hong Kong SAR China; ^2^ Department of Mechanical and Aerospace Engineering The Hong Kong University of Science and Technology Clear Water Bay Kowloon Hong Kong SAR China; ^3^ Department of Advanced Design and Systems Engineering City University of Hong Kong Kowloon Tong Kowloon Hong Kong SAR China

**Keywords:** flexible thermal sensor array, ionic thermoelectric hydrogel, polyquaternium‐10, Soret effect, thermopower

## Abstract

Ionic circuits using ions as charge carriers have demonstrated great potential for flexible and bioinspired electronics. The emerging ionic thermoelectric (iTE) materials can generate a potential difference by virtue of selective thermal diffusion of ions, which provide a new route for thermal sensing with the merits of high flexibility, low cost, and high thermopower. Here, ultrasensitive flexible thermal sensor arrays based on an iTE hydrogel consisting of polyquaternium‐10 (PQ‐10), a cellulose derivative, as the polymer matrix and sodium hydroxide (NaOH) as the ion source are reported. The developed PQ‐10/NaOH iTE hydrogel achieves a thermopower of 24.17 mV K^−1^, which is among the highest values reported for biopolymer‐based iTE materials. The high *p*‐type thermopower can be attributed to thermodiffusion of Na^+^ ions under a temperature gradient, while the movement of OH^−^ ions is impeded by the strong electrostatic interaction with the positively charged quaternary amine groups of PQ‐10. Flexible thermal sensor arrays are developed through patterning the PQ‐10/NaOH iTE hydrogel on flexible printed circuit boards, which can perceive spatial thermal signals with high sensitivity. A smart glove integrated with multiple thermal sensor arrays is further demonstrated, which endows a prosthetic hand with thermal sensation for human–machine interaction.

## Graphical abstract

This work demonstrates ultrasensitive flexible thermal sensor arrays based on the polyquaternium‐10/NaOH ionic thermoelectric (iTE) hydrogel. The selective thermal diffusion of ions in the iTE hydrogel leads to a high thermopower of 24.17 mV K^−1^. The thermal sensor array is able to perceive spatial thermal signals with high sensitivity, which endows a prosthetic hand with thermal sensation for human–machine interaction.

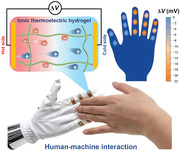

## 1 Introduction

Thermal sensation is an essential function of natural systems, which can perceive thermal stimuli from the surroundings and prevent potential thermal discomforts and risks. Similarly, artificial intelligent systems usually require temperature sensors to convert thermal information to measurable signals for further processing. However, due to their inherent rigidity, conventional thermistors and thermocouples cannot fulfill the requirements of flexible electronics and bioinspired systems, which lead to a growing demand for soft and deformable thermal sensing materials. Hydrogels have emerged as promising materials for flexible thermal sensors owing to their softness and biocompatibility. Recently, various hydrogel‐based thermal sensors based on resistive, capacitive, amperometric, or thermochromic sensing have been reported.^[^ ^1^, ^2^, ^3^, ^4^, ^5^ ^]^ However, as the resistance and capacitance of the hydrogels are also sensitive to strain, humidity, and gases,^[^ ^6^, ^7^, ^8^, ^9^ ^]^ in addition to temperature, it remains challenging to eliminate the potential interference from those signals for hydrogel‐based thermal sensors. The recent advance in ionic thermoelectric (iTE) materials^[^ ^10^, ^11^ ^]^ provides a new route for thermal sensing. Similar to the Seebeck effect^[^ ^12^, ^13^ ^]^ in electron‐based thermoelectric materials, iTE materials can generate a potential difference through the selective migration of ions under a temperature gradient, i.e., the so‐called Soret effect.^[^ ^14^, ^15^, ^16^ ^]^ Due to the high flexibility of polymer matrices, abundance of compositional elements, ease of sensing signal decoupling, and most importantly, ultrahigh thermopowers, iTE materials are promising thermal sensing materials for flexible and biocompatible systems. To date, remarkable thermopowers on the order of tens of mV K^−1^ have been reported for iTE materials,^[^ ^17^, ^18^, ^19^, ^20^, ^21^, ^22^, ^23^, ^24^, ^25^, ^26^, ^27^, ^28^, ^29^, ^30^, ^31^ ^]^ which is more than one order of magnitude higher than conventional thermoelectric materials.

The major strategy to achieve high thermopowers for iTE materials is to enlarge the difference in thermal mobility of cations and anions. Specifically, the ion–dipole^[^ ^17^, ^18^, ^19^ ^]^ and ion–ion^[^ ^21^, ^22^, ^23^, ^24^, ^25^, ^26^, ^27^, ^28^, ^29^, ^30^ ^]^ interactions between the polymer matrices and ion sources are commonly used to manipulate thermodiffusion of mobile ions. The ion–ion interaction exists between charged ends of polymers and mobile ions, which has been extensively applied in various polyelectrolytes, e.g., poly(ethylene oxide),^[^ ^22^ ^]^ polystyrene sulfonate,^[^ ^28^, ^29^ ^]^ polyurethane,^[^ ^30^ ^]^ etc. In contrast, the ion–dipole interaction occurs between charged ions and polymeric polar groups, such as hydroxyl and fluorinated groups. Poly(vinylidene fluoride‐*co*‐hexafluoropropylene) (PVDF‐HFP) is a typical polyelectrolyte endowed with the ion–dipole interaction, which has abundant fluorinated groups on the backbone and strong electron‐withdrawing capability. Zhao et al. developed a composite gel by incorporating an ionic liquid, 1‐ethyl‐3‐methylimidazolium bis(trifluoromethylsulfonyl)imide ([EMIM][TFSI]), into the PVDF‐HFP matrix, which achieved an *n*‐type thermopower of −4 mV K^−1^.^[^ ^17^ ^]^ Interestingly, it has been shown that the thermopowers of PVDF‐HFP‐based ionogels can be tuned over a wide range by introducing additives. Through the addition of polyethylene glycol, the movement of anions was deliberately confined, which led to a high *p*‐type thermopower of 14 mV K^−1^.^[^ ^17^ ^]^ Similarly, Liu et al. demonstrated tunable thermopowers from −15 to +17 mV K^−1^ under relative humidity (RH) of 60% for PVDF‐HFP‐based ionogels.^[^ ^18^ ^]^ Chi et al. developed ionogels by combining PVDF‐HFP with sodium bis(trifluoromethylsulfonyl)imide (NaTFSI) and propylene carbonate, and achieved thermopowers in a wide range from −6 to +20 mV K^−1^.^[^ ^19^ ^]^

Despite the significant advance in enhancing the ionic thermopowers, the synthetic polymer matrices used for the majority of iTE materials raise environmental concerns. It has drawn growing interest to develop green iTE materials based on biopolymers and their derivatives. With the advantages of abundance, low cost, and biocompatibility, biopolymers are ideal matrices for iTE materials in particular for the large‐scale production and wearable applications. Biopolymer derivatives such as gelatin^[^ ^21^ ^]^ and alginate^[^ ^26^ ^]^ have been used as the polymer matrices for iTE materials. It was reported that their negatively charged terminating groups (‐COO^−^) might facilitate the migration of cations through counterion condensation proposed by Manning.^[^ ^32^ ^]^ As the most abundant polymer on Earth, cellulose and its derivatives have also been explored for developing high‐performance iTE materials. Yang et al. reported a biocompatible hydrogel containing sodium carboxymethyl cellulose (CMC‐Na) with a thermopower of 17.1 mV K^−1^ and further demonstrated its application as an iTE supercapacitor.^[^ ^23^ ^]^ Liu et al. developed a bacterial‐cellulose‐based ionogel with good mechanical properties and a high thermopower of 18.04 mV K^−1^.^[^ ^31^ ^]^ Li et al. demonstrated highly selective ion transport in a cellulose membrane, where negative surface charges facilitated thermodiffusion of Na^+^ through the well‐aligned nanochannels but impeded the movement of OH^−^, resulting in an impressive thermopower of 24 mV K^−1^.^[^ ^25^ ^]^

In this work, we report a high‐performance and cost‐effective iTE hydrogel, which comprises polyquaternium‐10 (PQ‐10), a cellulose derivative, as the polymer matrix and sodium hydroxide (NaOH) as the ion source. The PQ‐10/NaOH iTE hydrogel was prepared by a facile dissolving and drying process. A thermopower of 24.17 mV K^−1^ is achieved for the developed iTE hydrogel with a PQ‐10 to NaOH weight ratio of 5:2 and a water content of ≈40 wt%. The high *p*‐type thermopower can be ascribed to thermodiffusion of hydrated Na^+^ ions under a temperature gradient, while the movement of OH^−^ ions is impeded by the strong electrostatic interaction with cationic polymer chains. Flexible thermal sensor arrays were developed based on the high‐thermopower PQ‐10/NaOH iTE hydrogel, which were able to detect the spatial temperature distribution with a sensitivity of 2.7 mV K^−1^. Moreover, a smart glove integrated with multiple thermal sensor arrays was further developed and applied on a prosthetic hand, which demonstrated the promising application of the PQ‐10/NaOH iTE hydrogel for artificial thermal sensing.

## 2 Results and Discussion

### 2.1 PQ‐10/NaOH iTE Hydrogels

**Figure 1:**
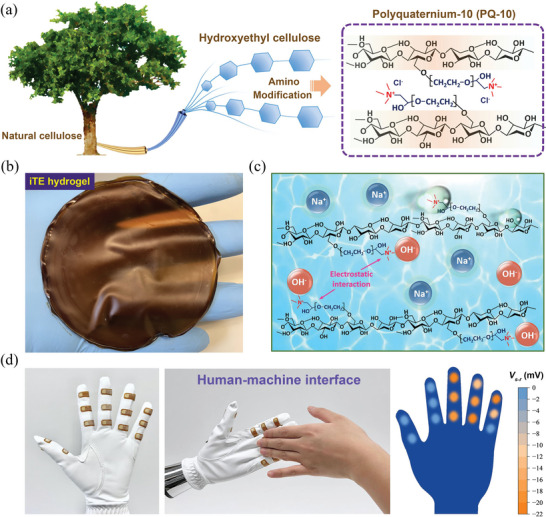
a) Chemical structure of PQ‐10 derived from natural cellulose. b) Photograph of the fabricated PQ‐10/NaOH iTE hydrogel. c) Schematic of the electrostatic interactions between PQ‐10 and OH^−^ anions in the developed iTE hydrogel. d) Smart glove integrated with multiple PQ‐10/NaOH thermal sensor arrays as the human–machine interface.

In this work, we developed an iTE hydrogel consisting of PQ‐10 as the polymer matrix and NaOH as the ion source. As a cationic hydroxyethyl cellulose derivative, PQ‐10 is typically produced through reacting hydroxyethyl cellulose with epichlorohydrin followed by quaternization with trimethylamine, as shown in **Figure** **1**a. PQ‐10 is chosen as the polymer matrix because its cationic polymer chains are likely to electrostatically attract anions and thus facilitate the selective thermodiffusion of cations. The iTE hydrogel (Figure 1b) was prepared by dissolving PQ‐10 and NaOH dry powders in deionized water, followed by drying the obtained solution under controlled conditions. Figure 1c schematically depicts the interactions among PQ‐10 polymer chains, water molecules, and mobile ions in the developed PQ‐10/NaOH iTE hydrogel. The hydroxyl and quaternary amino groups of PQ‐10 can form hydrogen bonds with water molecules, thus facilitating the dissolution of PQ‐10 in water. Moreover, the positively charged quaternary amino groups of PQ‐10 can attract OH^−^ anions through electrostatic interaction but repel Na^+^ cations, which may lead to the selective ion transport behavior. The thermally driven ion transport in the hydrogel can be a promising solution for thermal sensing, which endows a smart glove with thermal sensation as the human–machine interface (Figure 1d).

### 2.2 Ionic Thermoelectric Properties of the PQ‐10/NaOH iTE Hydrogels

**Figure 2:**
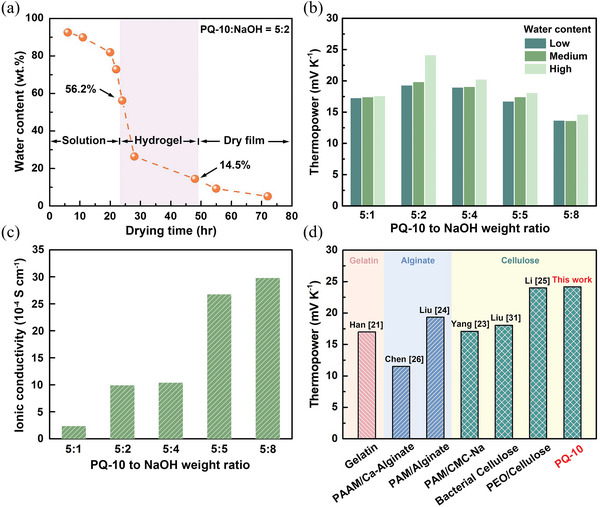
a) Variation of the water content with the drying time for the PQ‐10/NaOH solution/hydrogel/dry film with a PQ‐10 to NaOH weight ratio of 5:2. b) Thermopower of the developed PQ‐10/NaOH iTE hydrogels with different weight ratios and water contents. c) Ionic conductivity of the PQ‐10/NaOH iTE hydrogels with different weight ratios. d) Comparison of the thermopower of the PQ‐10/NaOH iTE hydrogel developed in this work with the absolute values of the state‐of‐art iTE materials based on biopolymer matrices.^[^ ^21^, ^23^, ^24^, ^25^, ^26^, ^31^ ^]^

Freestanding iTE hydrogels with a variety of PQ‐10 to NaOH weight ratios and water contents were prepared in this work. At a fixed loading of NaOH, the water content of the iTE hydrogel was controlled by tuning the drying time. **Figure** **2**a shows the variation of the water content with the drying time for the PQ‐10/NaOH solution or film with a PQ‐10 to NaOH weight ratio of 5:2. With the depletion of water, the sample morphology gradually changes from the prepared solution to a hydrogel film, and eventually a dry film. It is worth noting that too much water content cannot sustain enough mechanical strength for a freestanding film, while the dry film is detrimental for ion transport and also shows poor affinity with electrodes. Therefore, the water content of the developed iTE hydrogels was controlled in a proper range of 14.5–56.2% to maintain the gel‐like morphology and ensure that the film can be peeled off from the substrate.

The thermopower of the iTE hydrogel was characterized along the in‐plane direction with a homemade experimental setup (Figure S1, Supporting Information) under RH of 50–60%. Under a temperature gradient, a potential difference is generated between two ends of the hydrogel film due to thermodiffusion of mobile ions. During characterization, the temperatures at two ends of the iTE hydrogel film (*T*_H_ and *T*_C_) and the generated potential difference (*V*_H_  −  *V*_C_) were recorded. Similar to conventional thermoelectric materials, the thermopower of an iTE hydrogel is defined as $S\;=\;-\frac{V_{H}-V_{C}}{T_{H}-T_{C}}$,^[^ ^21^, ^33^ ^]^ which can be determined from the slope of the linear fitting curve of (*V*_H_  −  *V*_C_) versus (*T*_H_  −  *T*_C_). An iTE material with a negative thermopower is defined as an *n*‐type material, while the one with a positive thermopower is a *p*‐type material.^[^ ^21^ ^]^ As shown in Figure S2 in the Supporting Information, a negative potential difference (*V*_H_ − *V*_C_) was observed for a positive temperature difference (*T*_H_ − *T*_C_), corresponding to a positive thermopower. The positive sign of the thermopower indicates that the developed PQ‐10/NaOH iTE hydrogel is a *p*‐type material where Na^+^ cations are dominant charge carriers.

Figure 2b shows the thermopowers of the PQ‐10/NaOH iTE hydrogels with the PQ‐10 to NaOH weight ratio ranging from 5:1 to 5:8. At each weight ratio, the thermopower was measured at low, medium, and high water contents, respectively, by controlling the drying time of the iTE hydrogel. At a fixed weight ratio, a higher water content leads to a larger thermopower. The increase of the thermopower with water content can be attributed to the hydration interaction between Na^+^ ions and water molecules. A higher water content facilitates the hydration of Na^+^ ions and thus promotes the directional diffusion of cations under a temperature gradient. As shown in Figure 2b, for the similar water content, the thermopower first increases with the increase of NaOH loading, reaches the highest value at the weight ratio of 5:2, and then gradually decreases. The ionic conductivity of the developed hydrogels was characterized through electrochemical impedance spectroscopy (EIS). Figure 2c plots the ionic conductivity of the pristine PQ‐10 and PQ‐10/NaOH hydrogels with different weight ratios. With the increase of NaOH loading, a remarkable enhancement of the ionic conductivity was observed for the PQ‐10/NaOH iTE hydrogels, indicating the increase of mobile ions.^[^ ^19^ ^]^ The ionic conductivity of the iTE hydrogel with a weight ratio of 5:8 reaches 30 × 10^−4^ S cm^−1^, which is 20 times that of the pristine PQ‐10 hydrogel (1.5 × 10^−4^ S cm^−1^). However, the inhomogenous dispersion and aggregation of NaOH particles were observed for the samples with weight ratios of 5:5 and 5:8, suggesting that the PQ‐10 matrix is not able to accommodate high loading of NaOH (Figure S3, Supporting Information). The cyclic voltammetry (CV) curves of both the pristine PQ‐10 and PQ‐10/NaOH solutions exhibit nearly ideal rectangular shape without redox peaks (Figure S4, Supporting Information), which confirms that the ionic thermopower purely comes from thermodiffusion of mobile ions. The thermopower of the PQ‐10/NaOH iTE hydrogel with a weight ratio of 5:2 and a water content of ≈40 wt% reaches 24.17 mV K^−1^, which is among the highest values reported for biopolymer‐based iTE materials,^[^ ^21^, ^23^, ^24^, ^25^, ^26^, ^31^ ^]^ as shown in Figure 2d.

### 2.3 Underlying Mechanisms of the High Thermopower of the PQ‐10/NaOH iTE Hydrogel

**Figure 3:**
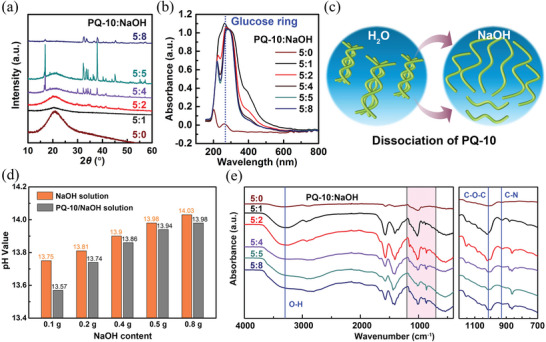
Characterization of the interactions among the polymer matrix, mobile ions, and water molecules in the PQ‐10/NaOH iTE hydrogels. a) XRD spectra of the PQ‐10/NaOH dry films with different weight ratios. b) UV‐vis spectra of the diluted PQ‐10/NaOH solutions with different weight ratios. c) Schematic of the facilitated dissociation of PQ‐10 polymer chains with the addition of NaOH. d) Comparison of the pH values of the NaOH solutions before and after the addition of 0.5 g PQ‐10 dry powders. e) FTIR spectra of the PQ‐10/NaOH dry films with different weight ratios.

To fully understand the high thermopower of the PQ‐10/NaOH iTE hydrogel, various materials characterization techniques were adopted to examine the interactions among the polymer matrix, mobile ions, and water molecules. The ion source NaOH is expected to facilitate the dissociation of PQ‐10 as alkali cations and OH^−^ anions can effectively break intermolecular hydrogen bonds of cellulose.^[^ ^34^, ^35^ ^]^ To confirm this, X‐ray diffraction (XRD) characterization was conducted on both the pristine PQ‐10 and PQ‐10/NaOH dry films. The pristine PQ‐10 film shows a strong diffraction peak at 2*θ* = 20° (**Figure** **3**a), corresponding to the crystalline plane (110) of cellulose II.^[^ ^36^ ^]^ With the addition of NaOH, the diffraction peak becomes less obvious, indicating the transformation of cellulose from the crystalline to amorphous phase. The diffraction peaks of NaOH crystals become dominant for the PQ‐10/NaOH films with weight ratios of 5:4, 5:5, and 5:8, which confirms that PQ‐10 cannot accommodate high loading of NaOH and the optimal weight ratio is 5:2 for uniform dispersion of ion source and good film morphology. The UV‐vis spectroscopy was further conducted to characterize the concentration of PQ‐10 polymer chains in the pristine PQ‐10 and PQ‐10/NaOH solutions. The characteristic peak of the glucose ring, i.e., a broad UV absorption band at 270 nm,^[^ ^37^ ^]^ is much stronger for the PQ‐10/NaOH solution compared to the pristine PQ‐10 solution, indicating that more PQ‐10 polymer chains are dissolved and hydrated with the presence of NaOH (Figure 3b). Both XRD and UV‐vis results suggest that NaOH facilitates the dissolution process of PQ‐10 through breaking the intermolecular hydrogen bonds between PQ‐10 polymer chains, as schematically shown in Figure 3c. The pristine PQ‐10 is only partially dissolved in deionized water, while the PQ‐10 bundles can be completely destroyed by NaOH, leading to the exposure of more polymer chains to water molecules.

PQ‐10 is a cationic polymer matrix with positively charged quaternary amino groups, which can potentially attract OH^−^ ions onto polymer chains through electrostatic interaction. The interaction between PQ‐10 and OH^−^ ions can be simply examined by the pH test. Upon adding 0.5 g PQ‐10 dry powders into the solutions with 0.1–0.8 g NaOH, the pH values of all solutions decrease (Figure 3d and Figure S5, Supporting Information), indicating the decrease of the OH^−^ concentration with the presence of PQ‐10. Fourier‐transform infrared (FTIR) spectroscopy was further conducted to disclose the intermolecular interactions in the PQ‐10/NaOH film. Figure 3e shows the spectral profiles of the pristine PQ‐10 and PQ‐10/NaOH dry films. With the addition of NaOH, the increase in the intensity of the peak at 3300 cm^−1^ can be attributed to –OH stretching vibration.^[^ ^38^ ^]^ The characteristic peaks of PQ‐10 lie at 1014 and 930 cm^−1^, corresponding to the C—O—C vibration^[^ ^39^ ^]^ and the C—N vibration for –N^+^‐(CH_3_)_3_ groups,^[^ ^40^ ^]^ respectively. The enhancement in the intensity of the C—N peak for the PQ‐10/NaOH films can be attributed to the strong electrostatic interaction between –N^+^‐(CH_3_)_3_ groups and OH^−^ ions. The pH and FTIR results reveal that PQ‐10 polymer chains can selectively pin OH^−^ anions on the quaternary amino sites through the electrostatic interaction in the PQ‐10/NaOH iTE hydrogel.

**Figure 4:**
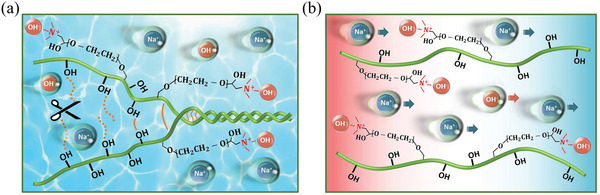
Schematics of the interactions in the PQ‐10/NaOH solution or hydrogel. a) Dissociation of PQ‐10 polymer chains facilitated by NaOH. b) Selective migration of Na^+^ cations in the iTE hydrogel under a temperature gradient.

**Figure** **4** schematically depicts the interactions among PQ‐10 polymer matrix, Na^+^ cations, OH^−^ anions, and water molecules in the PQ‐10/NaOH iTE hydrogel. As mentioned above, both Na^+^ cations and OH^−^ anions can break the hydrogen bonds between PQ‐10 polymer chains, leading to more cationic quaternary amino sites. The PQ‐10 polymer chains attract OH^−^ anions onto the quaternary amino sites through electrostatic interaction, while Na^+^ cations can move freely under a temperature gradient, resulting in high *p*‐type thermopower for the PQ‐10/NaOH iTE hydrogel. A high water content in the iTE hydrogel will facilitate the hydration of Na^+^ cations and increase their thermal mobility.

### 2.4 A Flexible Thermal Sensor Array based on the PQ‐10/NaOH iTE Hydrogel

**Figure 5:**
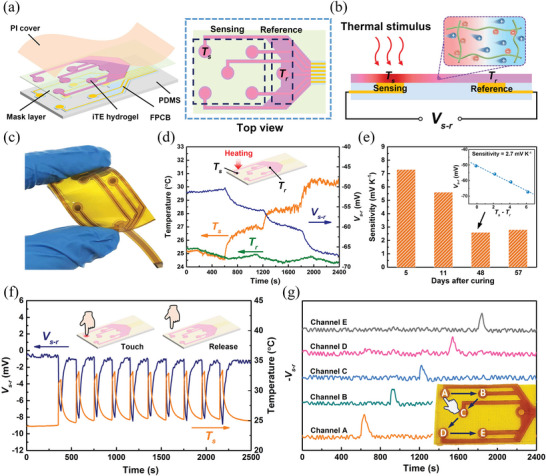
a) Structure of the flexible thermal sensor array based on the PQ‐10/NaOH iTE hydrogel. b) Schematic of the sensing mechanism based on the high thermopower of the PQ‐10/NaOH iTE hydrogel. c) Photograph of the fabricated flexible thermal sensor array. d) Recorded *V*_s − r_, *T*_s_, and *T*_r_ when a step thermal stimulus is applied to a single channel at Day 48 after curing. e) Variation of the sensitivity with the days after curing. The inset shows the determination of the sensitivity through linearly fitting the *V*_s − r_ versus (*T*_s_ − *T*_r_) curve. f) Cyclic finger touch‐release test for a single channel. g) Sequential finger touch‐release test for multiple channels of the sensor array. The inset shows the touch sequence from A to E.

The developed PQ‐10/NaOH iTE hydrogel is promising for thermal sensing due to high thermopower, good flexibility, and ease of fabrication. A flexible thermal sensor array was fabricated based on the PQ‐10/NaOH iTE hydrogel with a weight ratio of 5:2. The iTE hydrogel was patterned onto a customized flexible printed circuit board (FPCB) consisting of five sensing electrodes uniformly distributed in a 3 cm × 3 cm sensing region and a common reference electrode, as schematically shown in **Figure** **5**a. The top surface of the sensor array was covered by a polyimide (PI) tape to reduce the interference from the ambient environment such as humidity, gases, etc. The sensing and reference electrodes were connected to a data logger (Agilent, 34970A) through a multichannel connector. Figure 5b illustrates the working principle of the developed ionic thermal sensor. Once a thermal stimulus is exerted on a sensing node, a potential difference (*V*_s − r_) will be generated in the hydrogel between the sensing and reference electrodes due to the unbalanced diffusion of cations and anions under a temperature gradient, which will reveal the thermal condition in the sensing region accordingly.

Figure 5c shows a photograph of the fabricated flexible thermal sensor array. The sensitivity of a single channel of the sensor array was characterized through exerting a step change in temperature on the sensing node and monitoring the variation of *V*_s − r_. Figure 5d shows the measured *V*_s − r_ and the sensing (*T*_s_) and reference temperatures (*T*_r_) recorded by type K thermocouples during the radiative heating of a single sensing node. The characterization was conducted in an open indoor environment with a temperature of ≈25 °C. The change in the measured *T*_r_ is due to the fluctuation in the ambient temperature. With the step increase of *T*_s_, *V*_s − r_ shows a step decrease accordingly. The sensitivity of the sensing node can be determined from the slope of the linear fitting curve of *V*_s − r_ versus (*T*_s_ − *T*_r_) as shown in the inset of Figure 5e. As mentioned above, the thermopower of the PQ‐10/NaOH iTE hydrogel varies with water content. It is expected that the sensitivity of the sensing node will be affected by the loss of water content in the hydrogel over the time. As shown in Figure 5e, the sensitivity of the sensing node gradually decreases from 7.3 mV K^−1^ at Day 5 after curing to a stable value of 2.7 mV K^−1^ at Day 57. The water retention ability of the iTE hydrogel may be further improved through solvent replacement, i.e., partially replacing water with organic solvents,^[^ ^1^ ^]^ to maintain a high sensitivity over a long time. Nevertheless, even at more than 50 days after curing, the sensitivity of our hydrogel‐based thermal sensor is still more than one order of magnitude higher than those of commercial thermocouples (<0.1 mV K^−1^).^[^ ^41^ ^]^ Commercial thermocouples typically have a temperature resolution of 0.1 K.^[^ ^42^ ^]^ It is expected that our thermal sensor can detect a minimum temperature difference of 0.01 K due to higher sensitivity.

The capability of the developed sensor array for thermal sensing was demonstrated by a finger touch‐release test. When a sensing node is tapped by a finger, body heat will increase the temperature of the sensing node. During testing, the potential difference between the sensing and reference electrodes was recorded by a data logger (Agilent, 34970A), while the real‐time temperature of the sensing node was monitored by a type K thermocouple. Figure 5f shows the potential difference and the temperature of the sensing node recorded during a cyclic finger touch‐release test. In response to the temperature stimulus exerted by a finger, the recorded potential difference shows an instantaneous drop at the touching moment and quickly rebounds once the finger is retracted from the sensing node. There is a good agreement between the potential difference and the temperature stimulus, indicating high repeatability of our thermal sensor.

**Figure 6:**
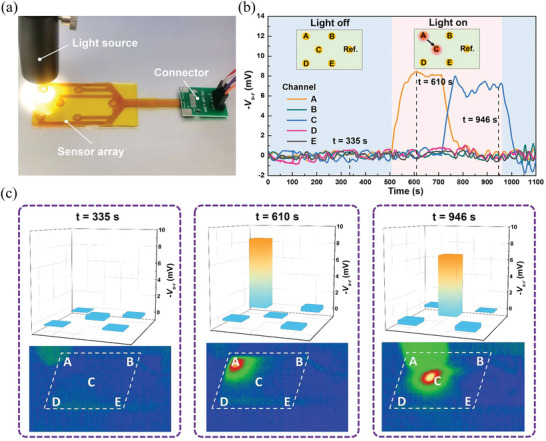
a) Experimental setup for characterizing the spatial thermal sensing function of the flexible thermal sensor array under the noncontact thermal stimulus from a light source. b) Responses of the flexible thermal sensor array when the sensing nodes A and C were illuminated by the light source. c) Voltage distribution and thermal mapping of the thermal sensor array at three representative moments.

The spatial thermal sensing function of the sensor array was characterized with contact and noncontact thermal stimuli, respectively. The sensing nodes from A to E were tapped by a finger sequentially, as shown in the inset of Figure 5g. During testing, the potential differences between all sensing electrodes and the reference electrode were simultaneously recorded. As shown in Figure 5g, when a specific sensing node was touched by a finger, the potential difference between the corresponding sensing electrode and the reference electrode shows an obvious peak, while the signals remain nearly unchanged for other channels. As a result, the time and location of the finger contact with the sensor array can be simply determined through simultaneously monitoring the signals of all channels. In the noncontact mode, a certain region of the sensor array was exposed to a light source and consequently the surface was heated up by thermal radiation (**Figure** **6**a). During testing, the signals of all sensing nodes were recorded and the surface temperature distribution was monitored by a thermal imaging camera (H10, HIKVISION). Figure 6b shows the recorded real‐time voltages of all sensing nodes, while the voltage distribution and thermal mapping at three representative moments are shown in Figure 6c. When the light source is off (*t* = 335 s), both the voltage and the surface temperature show a uniform distribution. When the top left corner (*t* = 610 s) or the center (*t* = 946 s) of the sensor array was exposed to the light source, a much larger signal was observed for the corresponding sensing node (A or C) compared to other sensing nodes, which was consistent with the thermal images captured by the IR camera. The ultrasensitive flexible thermal sensor array based on the PQ‐10/NaOH iTE hydrogel demonstrates the promising performance to perceive the spatial thermal stimulus, such as contact with a finger or exposure to thermal radiation.

### 2.5 A Smart Glove with Spatial Thermal Perception

**Figure 7:**
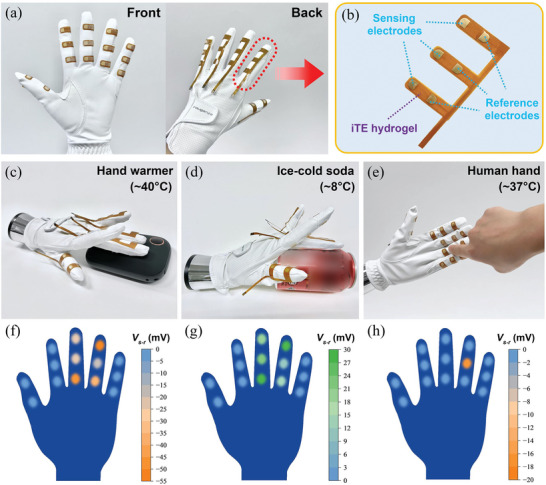
a) Photographs of a smart glove integrated with multiple thermal sensor arrays. b) Photograph of the flexible thermal sensor array attached onto each finger. Demonstration of the thermal sensation function of the smart glove when in contact with c,f) a hand warmer, d,g) an ice‐cold soda can, and e,h) a human hand.

A smart glove integrated with multiple PQ‐10/NaOH thermal sensor arrays was further developed to demonstrate the potential application of the iTE hydrogel in artificial perception systems. To mimic the thermal sensation of the human skin, 14 sensing nodes are distributed among five fingers of the glove, as shown in **Figure** **7**a. Each finger is wrapped with a thermal sensor array (Figure 7b), where the sensing and reference electrodes are attached to the front and back sides of the finger, respectively (Figure 7a). The glove was worn on a prosthetic hand and tested through touching various objects with different temperatures (Figure 7c–e).

Figure 7f–h presents the voltage responses of the smart glove when the prosthetic hand is in contact with a hand warmer (≈40 °C), an ice‐cold soda can (≈8 °C), and a human hand (≈37 °C), respectively, as shown in Figure 7c–e. The ambient temperature was kept at ≈23 °C during testing. Upon touching a hot/cold object, negative/positive voltages were recorded for the sensing nodes in contact with the object, while the voltages for other sensing nodes remained nearly zero (Figure 7f–h), corresponding to the temperature distribution on the smart glove. We note that the generated voltages of the sensor arrays are purely due to the temperature but not pressure stimuli as no voltage change is recorded when a room‐temperature weight is placed onto the sensing node (Figure S6, Supporting Information). Through monitoring the voltages of all sensing nodes, the smart glove is able to perceive the temperature and touching position of the object, demonstrating the potential application of the flexible thermal sensor array for the intelligent interaction between robots and their environment.

## 3 Conclusions

In this work, we report a high‐thermopower and cost‐effective PQ‐10/NaOH iTE hydrogel and ultrasensitive hydrogel‐based flexible thermal sensor arrays. The iTE hydrogel is free of high‐cost raw materials (e.g., ionic liquids) and does not involve any complex synthesis route (e.g., synthesis of the synthetic polymer, chemical crosslinking, etc.). The developed PQ‐10/NaOH iTE hydrogel achieves a thermopower of 24.17 mV K^−1^ with a PQ‐10 to NaOH weight ratio of 5:2 and a water content of 40 wt%. The high *p*‐type thermopower originates from the electrostatic interaction between the polycationic polymer matrix and OH^−^ anions, leading to much higher thermal mobility of hydrated Na^+^ cations than OH^−^ anions. A higher water content is found to facilitate the hydration and diffusion of Na^+^, thus favoring a larger thermopower. An ultrasensitive flexible thermal sensor array was developed based on the PQ‐10/NaOH iTE hydrogel, which could maintain a sensitivity of 2.7 mV K^−1^ after nearly 2 months at the ambient conditions. In both contact and noncontact modes, the thermal sensor array can detect the real‐time spatial temperature distribution on the sensing surface. We further show that a smart glove integrated with multiple thermal sensor arrays can successfully detect the temperature and touching position of the object in contact, which demonstrates a great potential of the PQ‐10/NaOH iTE hydrogel to perceive thermal signals as the electronic skin.

## 4 Experimental Section

### Materials

NaOH was purchased from Dieckmann (Hong Kong) Chemical Industry Co., Ltd. PQ‐10 was obtained from Wuhan Lullaby Pharmaceutical Chemical Co., Ltd. All chemicals were used without further purification. Deionized water was purified using a Milli‐Q Direct 8 ultrapure water system (MilliporeSigma).

### Preparation of the PQ‐10/NaOH iTE Hydrogels

A certain amount of NaOH (e.g., 0.1–0.8 g) was first dissolved in 15 mL deionized water. Then, 0.5 g PQ‐10 dry powders were added into the as‐prepared NaOH solution under magnetic stirring. The solution was stirred at room temperature for at least 12 h until a homogenous solution was formed. The PQ‐10/NaOH solution was poured into a petri dish and then dried in an oven at 40 °C. The water content in the PQ‐10/NaOH iTE hydrogel can be tuned by controlling the drying time. The weight of the PQ‐10/NaOH solution or hydrogel was recorded from time to time (e.g., 2, 5, 10, 20 h after the start of the drying process) until the film was completely dried out, which could be used to determine the water content in the iTE hydrogel for a certain drying time. Once the water content is lower than ≈56 wt%, the PQ‐10/NaOH iTE hydrogel can be peeled off from the petri dish as a freestanding film.

### Ionic Thermoelectric Properties Characterization

The thermopower of the PQ‐10/NaOH iTE hydrogel was characterized through a homemade experimental setup under RH of 50–60%. As schematically shown in Figure S1 in the Supporting Information, two graphite electrodes were fixed on top of a glass substrate by silver paste, while two Peltier modules were adhered to the backside of the glass substrate for generating a temperature difference across the sample. The whole setup was mounted on top of a large copper block using thermal grease to ensure thermal stability during characterization. The iTE hydrogel was cut into a rectangular strip and placed bridging two graphite electrodes. The sample surface was covered with a piece of PI tape to attenuate the effect of the ambient environment on the thermopower measurement. During characterization, a DC current was passed through two Peltier modules by using a SourceMeter (Keithley 2425), which led to heating at one end and cooling at the other end of the sample. The temperatures at the hot and cold ends of the sample (*T*_H_ and *T*_C_) were measured by two type K thermocouples taped onto the top surface of graphite electrodes. The potential difference between two ends of the sample was recorded through two graphite electrodes connected to a data logger (Agilent 34970A). The temperature difference across the sample could be adjusted by tuning the current applied to the Peltier modules. For each temperature difference, the temperatures at two ends of the sample and the potential difference were continuously measured until reaching steady state.

For the ionic conductivity characterization, the PQ‐10/NaOH iTE hydrogel film was sandwiched between two stainless steel electrodes in a 2025 coin cell module,^[^ ^38^ ^]^ as schematically shown in Figure S7a in the Supporting Information, and assembled by using a crimper. The ionic resistance of the sample was characterized through EIS with a voltage amplitude of 5 mV and the frequency decreasing from 1 MHz down to 10 mHz. The ionic resistance of each sample was obtained from the intercept of the straight line on the abscissa in Figure S7b in the Supporting Information. The ionic conductivity (*σ*) of the PQ‐10/NaOH iTE hydrogel film could be determined by *σ*  =  *t*/(*RA*), where *t* is the film thickness, *R* is the measured ionic resistance, and *A* is the area of the sample.

### Materials Characterization

Materials characterization was conducted on the prepared solutions and dry films. The pH values of the solutions were measured using a pH meter (Eutech PC 700). The CV profiles were measured with an electrochemical workstation (BioLogic VMP3) for a potential range from −0.2 to 0.2 V at a scan rate of 10 mV s^−1^. The UV‐vis characterization was conducted with LabRAM HR800. All the solutions were diluted 50 times for the UV‐vis characterization. The hydrogel films were completely dried out in an oven to get rid of the influence of water. The XRD characterization (Rigaku, SmartLab) was performed on both PQ‐10 and PQ‐10/NaOH dry films to examine the sample crystallinity. The FTIR spectroscopy characterization (Bruker, Alpha) was conducted on the PQ‐10/NaOH dry films to investigate the interactions between ions and functional groups of PQ‐10.

### Fabrication of the Flexible Thermal Sensor Array

The fabrication process of a flexible thermal sensor array is schematically shown in Figure S8 in the Supporting Information. An FPCB with six electrodes was first prepared, among which five electrodes were patterned in the sensing region while the other one was located in the reference region. The FPCB was placed on a polydimethylsiloxane substrate. Then, a laser‐cut PI tape with the designed pattern was aligned with the FPCB and attached to the top surface as a mask layer. Next, the prepared PQ‐10/NaOH solution was drop casted onto the exposed area of the PI tape and dried in the ambient environment for 2 days. Finally, the top surface of the whole sensor array including the sensing and reference regions was covered by a PI tape for packaging. The flexible thermal sensor arrays for the smart glove (Figure 7a,b) were fabricated through a similar process.

## Conflict of Interest

The authors declare no conflict of interest.

## Supporting information

Supporting Information

## Data Availability

The data that support the findings of this study are available from the corresponding author upon reasonable request.
