# Supplementary material for: Ultrasensitive Flexible Thermal Sensor Arrays based on High‐Thermopower Ionic Thermoelectric Hydrogel
Source: Adv Sci (Weinh). 2023 Jul 3;10(25):2302685. doi: 10.1002/advs.202302685 (PMC10477880; doi:10.1002/advs.202302685)
Supplement: Supplementary file 1 — Supporting Information [file ADVS-10-2302685-s001.pdf]

ADVANCED SCIENCE
Open Access

Supporting Information

for *Adv. Sci.*, DOI 10.1002/advs.202302685

Ultrasensitive Flexible Thermal Sensor Arrays based on High-Thermopower Ionic Thermoelectric Hydrogel

*Yang Han, Haoxiang Wei, Yanjun Du, Zhigang Li, Shien-Ping Feng, Baoling Huang and Dongyan Xu**

Supporting Information

for

Ultrasensitive Flexible Thermal Sensor Arrays Based on the High-thermopower Ionic Thermoelectric Hydrogel

Yang Han, Haoxiang Wei, Yanjun Du, Zhigang Li, Shien-Ping Feng, Baoling Huang, Dongyan Xu*

Y. Han, H. Wei, Y. Du, D. Xu

Department of Mechanical and Automation Engineering, The Chinese University of Hong Kong, Shatin, New Territories, Hong Kong Special Administrative Region, China

E-mail: dyxu@mae.cuhk.edu.hk

Z. Li, B. Huang

Department of Mechanical and Aerospace Engineering, The Hong Kong University of Science and Technology, Clear Water Bay, Kowloon, Hong Kong Special Administrative Region, China

S.-P. Feng

Department of Advanced Design and Systems Engineering, City University of Hong Kong, Kowloon Tong, Kowloon, Hong Kong Special Administrative Region, China

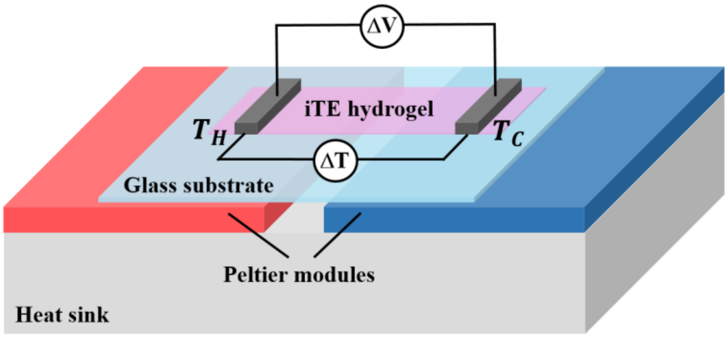

Fig. S1. Home-made experimental setup for characterizing the thermopower of the iTE hydrogel.

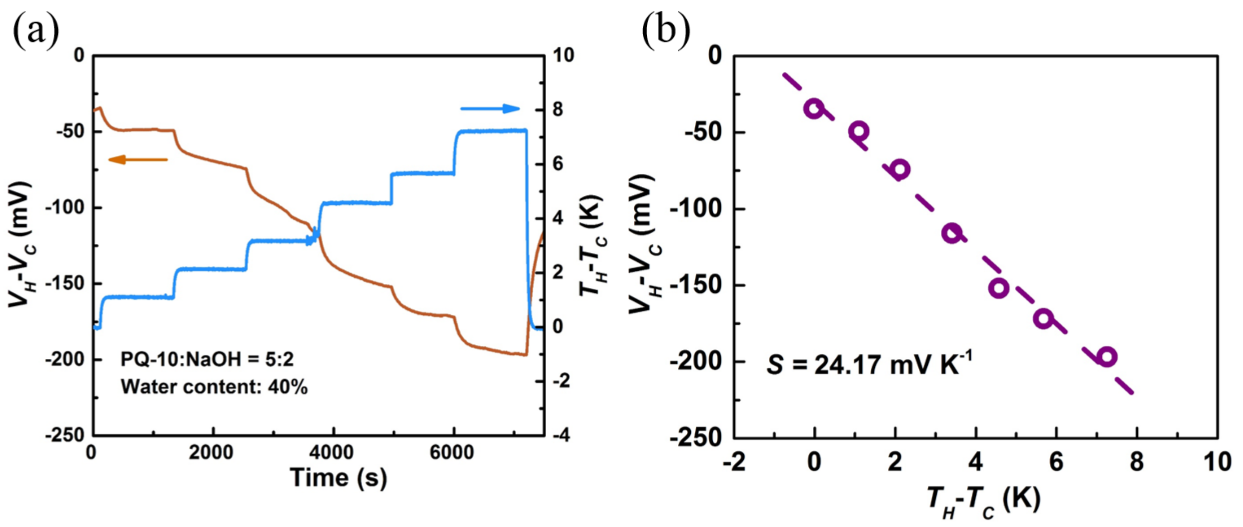

Fig. S2. (a) Measured potential and temperature differences for the PQ-10/NaOH iTE hydrogel with a weight ratio of 5:2 and a water content of 40 wt.%. (b) $(V_H - V_C)$ vs. $(T_H - T_C)$ curve for the raw data in (a). The thermopower of the iTE hydrogel can be determined from the slope of the linear fitting curve.

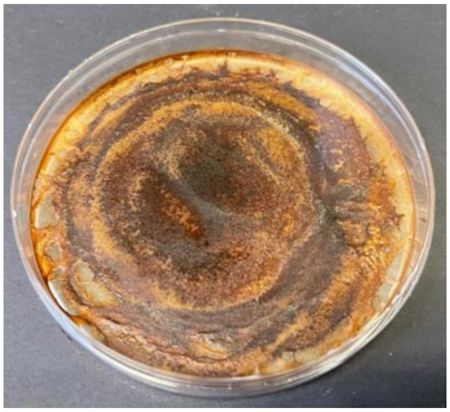

Fig. S3. Photograph of the PQ-10/NaOH iTE hydrogel with a weight ratio of 5:5.

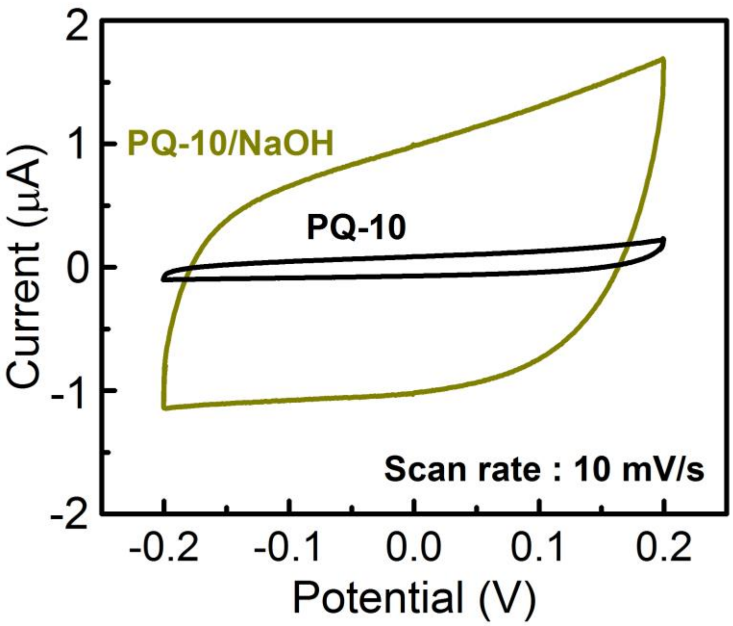

Fig. S4. CV curves of the pristine PQ-10 and PQ-10/NaOH solutions.

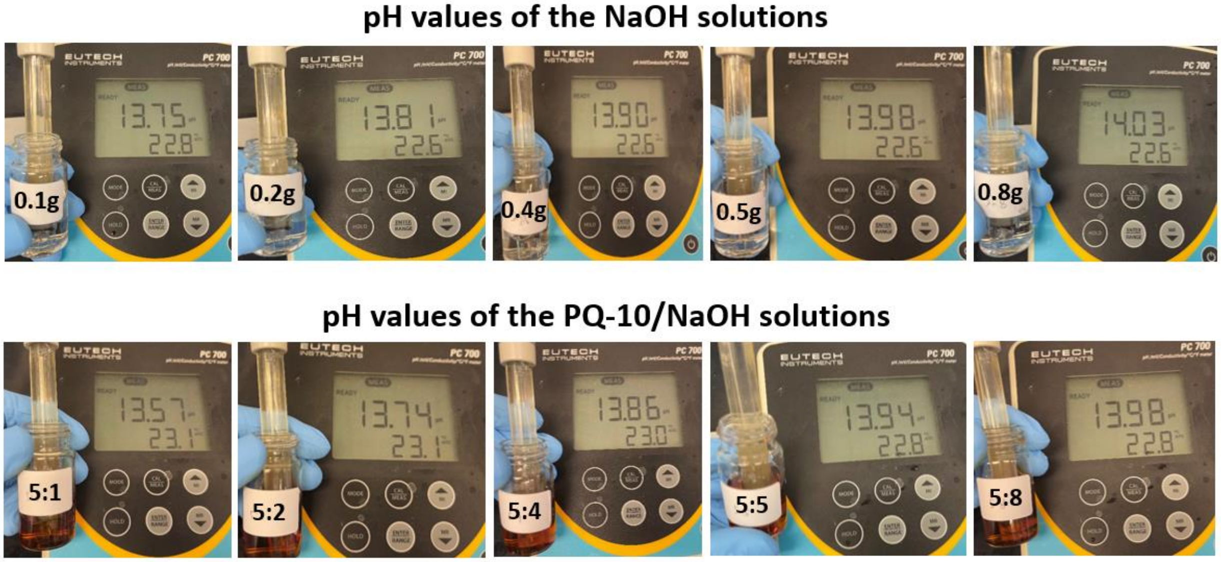

Fig. S5. Photographs of the pH value tests for the NaOH solutions before and after the addition of 0.5 g PQ-10 dry powders.

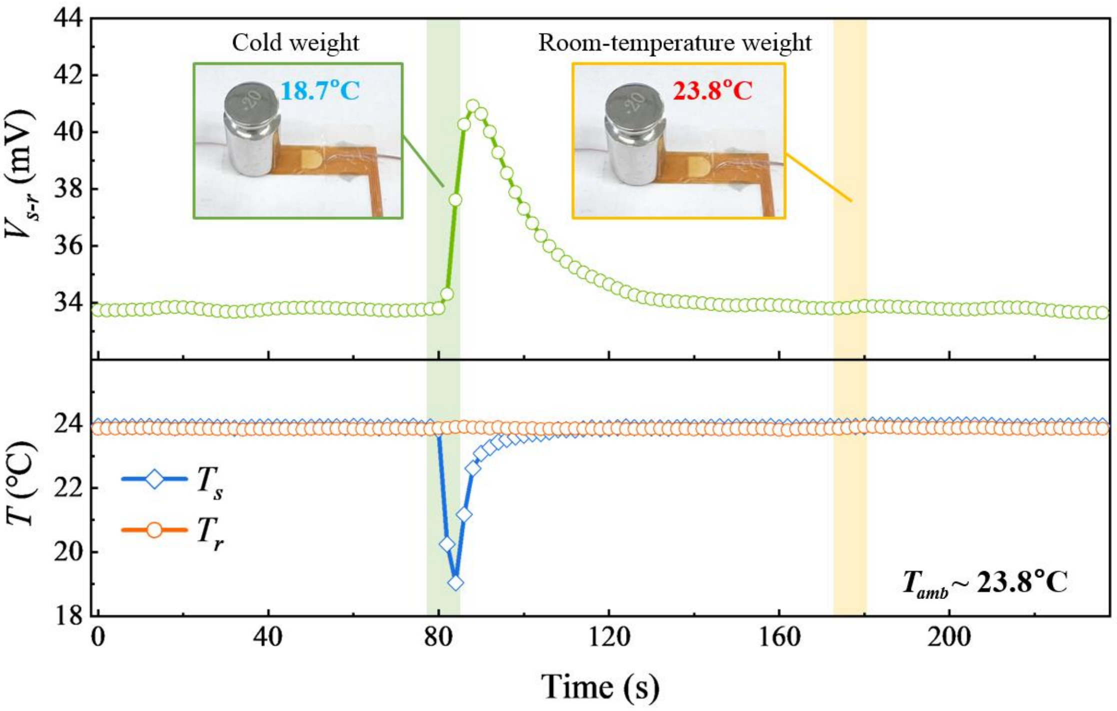

Fig. S6. Recorded voltages, sensing and reference temperatures when a cold or room-temperature weight was placed on the sensing node.

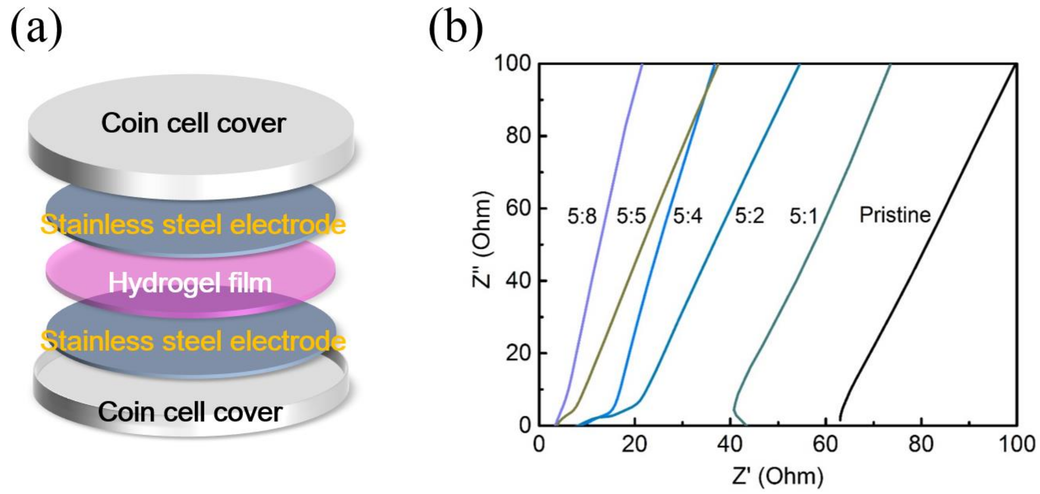

Fig. S7. (a) Schematic of the coin cell module for the EIS characterization. (b) EIS profiles of the pristine PQ-10 and PQ-10/NaOH hydrogel films with different weight ratios. The ionic resistance of each sample can be obtained from the intercept of the straight line on the abscissa.

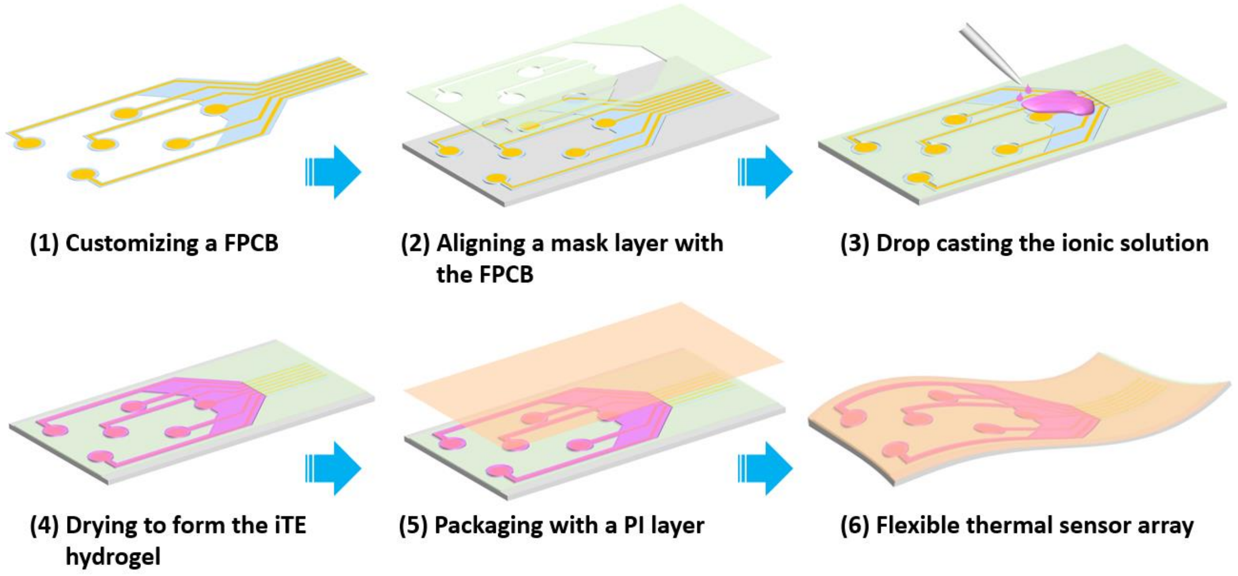

Fig. S8. Fabrication process of the flexible thermal sensor array.
